# Antagomir-*17-5p* Abolishes the Growth of Therapy-Resistant Neuroblastoma through p21 and BIM

**DOI:** 10.1371/journal.pone.0002236

**Published:** 2008-05-21

**Authors:** Laura Fontana, Micol E. Fiori, Sonia Albini, Loredana Cifaldi, Serena Giovinazzi, Matteo Forloni, Renata Boldrini, Alberto Donfrancesco, Valentina Federici, Patrizio Giacomini, Cesare Peschle, Doriana Fruci

**Affiliations:** 1 Department of Hematology, Oncology and Molecular Medicine, Istituto Superiore di Sanità, Rome, Italy; 2 Research Center, Ospedale Pediatrico Bambino Gesù, Rome, Italy; 3 Division of Pathology, Ospedale Pediatrico Bambino Gesù, Rome, Italy; 4 Division of Pediatric Oncology, Ospedale Pediatrico Bambino Gesù, Rome, Italy; 5 Pathology Laboratory, Regina Elena Cancer Institute, Rome, Italy; 6 Laboratory of Immunology, Regina Elena Cancer Institute, Rome, Italy; 7 IRCCS MultiMedica, Milan, Italy; Lehigh University, United States of America

## Abstract

We identified a key oncogenic pathway underlying neuroblastoma progression: specifically, MYCN, expressed at elevated level, transactivates the miRNA *17-5p-92* cluster, which inhibits p21 and BIM translation by interaction with their mRNA 3′ UTRs. Overexpression of miRNA *17-5p-92* cluster in *MYCN*-not-amplified neuroblastoma cells strongly augments their *in vitro* and *in vivo* tumorigenesis. *In vitro* or *in vivo* treatment with antagomir-*17-5p* abolishes the growth of *MYCN*-amplified and therapy-resistant neuroblastoma through p21 and BIM upmodulation, leading to cell cycling blockade and activation of apoptosis, respectively. In primary neuroblastoma, the majority of cases show a rise of miR-*17-5p* level leading to p21 downmodulation, which is particularly severe in patients with *MYCN* amplification and poor prognosis. Altogether, our studies demonstrate for the first time that antagomir treatment can abolish tumor growth *in vivo*, specifically in therapy-resistant neuroblastoma.

## Introduction

MicroRNAs (miRNAs or miRs) are conserved ∼22 nucleotide non-coding RNAs: they repress protein expression at post-transcriptional level[1]–[3], mainly by annealing with the 3′ UTR of the target mRNA, thus interfering with its translation and/or stability[4]. MiRNAs play important roles in the regulation of basic cell functions, including proliferation, differentiation and apoptosis[5]–[8]. Importantly, oncogenesis has been linked to deregulated expression of miRNAs, which act as tumor suppressors or oncomirs[9], [10] and may contribute to tumor invasion[11].

The miRNA *17-5p-92* cluster comprises seven miRNAs, transcribed as a polycistronic unit, and is homologous to the miRNA *106a-92* cluster[12]. We have previously shown that miR-*17-5p*, -*20a* and -*106a* regulate monocytopoiesis through *AML1* targeting[7]. Growing evidence documents deregulation of the miRNA *17-5p-92* cluster in cancer. Specifically, a genomic region containing the miRNA *17-5p-92* polycistron is amplified in B-cell lymphomas[13]. Furthermore, transcription of miRNA *17-5p-92* cluster is regulated by the oncogene c-myc[14], while its overexpression can contribute to tumorigenesis[15]–[18].

Neuroblastoma, accounting for 8–10% of pediatric tumors, originates from precursor cells of the peripheral nervous system. The most aggressive neuroblastomas are characterized by diverse genetic aberrations, including *MYCN* amplification, chromosome 1p deletion and unbalanced gain of chromosome 17q[19]. *MYCN* amplification occurs in 25% of the cases and correlates to both an aggressive phenotype and treatment failure[19]. The neuroblastoma progression linked to *MYCN* amplification, although well documented, is mediated by unknown molecular mechanisms. Some miRNAs, including miR-*9*, miR-*125* and miR-*34a* control neuroblastoma cell proliferation *in vitro* [20], [21], but their function has not been linked to neuroblastoma carrying *MYCN* amplification. p21^Cip1/Waf1/Sdi1^ (hereafter referred to as p21), the founding member of the Cip/Kip family of cyclin-dependent kinase (CDK) inhibitors, negatively regulates cell cycle progression by inhibiting a broad range of cyclin/Cdk complexes[22]. Specifically, p21 prevents cell cycle progression from G1 to S phase by inactivating the Cdk2-cyclin E complexes, that, in turn, inhibit the tumor suppressor protein retinoblastoma (pRb) required for entering S-phase[23]. It can also act as a tumor suppressor, as demonstrated by the higher susceptibility of *p21*-deficient mice to develop spontaneous tumors[24]. In spite of this, genetic alterations of p21 are rare in human tumor samples[25], suggesting that its oncogenic function is mostly mediated by a deregulated expression.

BIM (Bcl-2 interacting mediator of cell death) is one of the most potent pro-apoptotic BH3-only proteins: it binds to all pro-survival Bcl-2 family members with high affinity[26], [27], thereby releasing Bax or Bak proteins, the critical downstream effectors of the Bcl-2-dependent pathway of apoptosis[28]. *BIM* is a tumor suppressor gene, as demonstrated by the accelerated Myc-induced lymphomagenesis in Eµ-*myc* mice lacking *BIM* [29] and increased tumorigenesis of *BIM*-/- epithelial cells[30] .

In this study, we have investigated the molecular mechanisms underlying MYCN-induced neuroblastoma progression. Our findings indicate that an enhanced MYCN level, linked or not to *MYCN* amplification, transactivates the miRNA *17-5p-92* cluster at transcriptional level. The upmodulation of miR-*17-5p* mediates the oncogenic properties of *MYCN*, through a direct suppression of *p21* and *BIM* translation. Of particular interest is that treatment of *MYCN*-amplified neuroblastoma with antagomir-*17-5p* can abolish tumor growth, not only *in vitro* but also *in vivo*.

## Results

### MYCN transcriptionally regulates the expression of miRNA *17-5p-92* cluster in neuroblastoma

Transcription of the miRNA *17-5p-92* cluster is induced by the oncogene c-Myc[14]. Since c-Myc and MYCN share some transcriptional target, we hypothesized that MYCN may transactivate this cluster.

**Figure 1:**
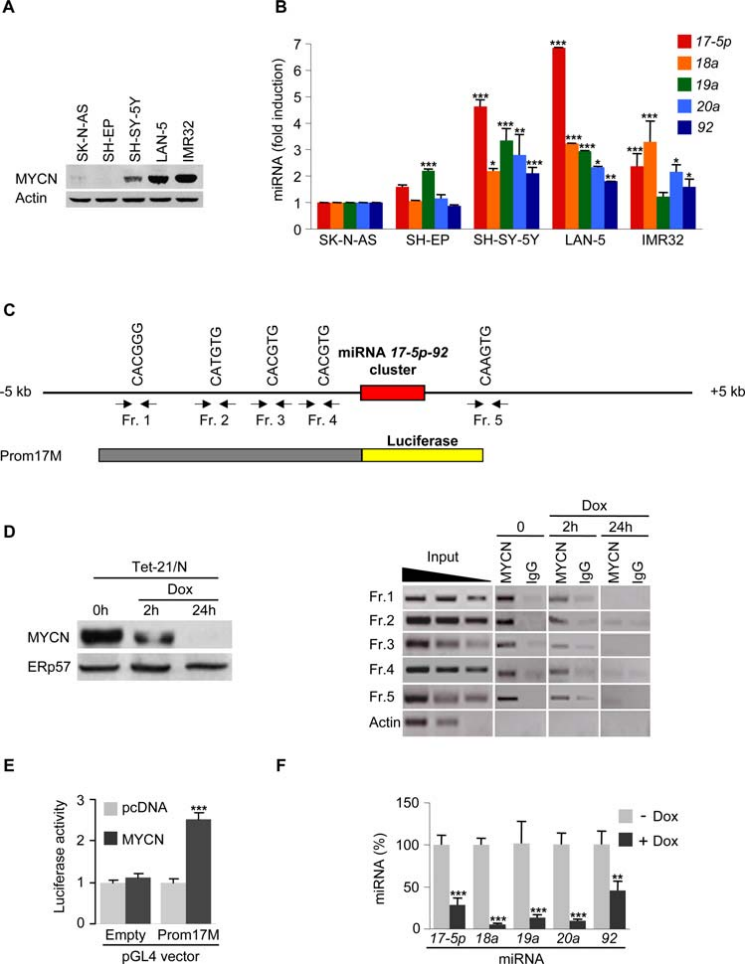
MYCN transactivates the miRNA *17-5p-92* cluster in neuroblastoma by directly binding to its promoter. (A) Western blot of MYCN in different neuroblastoma cell lines. A representative experiment is shown. (B) miRNA qRT-PCR analysis of miRNAs pertaining to the miRNA *17-5p-92* cluster in different neuroblastoma cell lines. The level of each miRNA is normalized to its expression in SK-N-AS cells (set as 1). Mean±s.d. (*n = 3*). (C) Schematic representation of the genomic region encompassing the miRNA *17-5p-92* cluster. Five putative MYCN binding sites (included in fragments 1, 2, 3, 4, 5) are indicated. (D) *Left panel*. Representative Western blot of MYCN in Tet-21/N cells untreated (0 h) or treated with doxycycline (Dox) for 2 or 24 h. ERp57 was used for normalization. *Right panel*. Chromatin immunoprecipitation with an anti-MYCN or a control anti-IgG antibodies on lysates from Tet-21/N cells untreated or treated with doxycycline for the indicated times. Control amplifications were carried out on either chromatin before immunoprecipitation (Input) or immunoprecipitated chromatin with oligonucleotides amplifying the β-*actin* gene (Actin). (E) Promoter assay with the pGL4Prom17M construct containing a 3731 bp fragment of the miRNA *17-5p-92* cluster promoter upstream the luciferase gene (indicated in C). SH-EP cells were transfected with pGL4 vector (Empty) or pGL4Prom17M (Prom17M) in combination with pcDNA3 (pcDNA) or piRV-neoSV-MYCN and luciferase activity was measured 72 h post-transfection. The bars represent the normalized luciferase activity (mean±s.d., *n = 5*). (F) miRNA qRT-PCR analysis of miRNAs pertaining to the miRNA *17-5p-92* cluster in Tet-21/N untreated (− Dox) or treated with doxycycline for 96 h (+ Dox). The level of each miRNA is reported as percentage of its expression in untreated cells (set as 100%). Mean±s.d. (*n = 3*). * P<0.05, ** P<0.01, *** P<0.001.

To verify this hypothesis, we first analyzed the expression of the miRNA *17-5p-92* cluster in five neuroblastoma cell lines expressing MYCN at either low level (SH-EP and SK-N-AS) or high level due to *MYCN* overexpression (SH-SY-5Y) or amplification (LAN-5 and IMR32) (Figure 1A). Most miRNAs encoded by the miRNA *17-5p-92* cluster were expressed at higher levels in cells overexpressing MYCN or carrying *MYCN* amplification, as evaluated by both Northern blot and qRT-PCR (Figure 1B and data not shown).

Thereafter, analysis of the genomic region surrounding the miRNA *17-5p-92* cluster for putative MYCN binding sites (including the canonical E-box CACGTG and the non-canonical sequences CATGTG, CACGGG and CAAGTG) revealed the presence of five MYCN putative binding sites, four upstream and one downstream the cluster (Figure 1C). To demonstrate the interaction between MYCN and the cluster promoter, chromatin immunoprecipitation (ChIP) experiments were performed using the Tet-21/N cell line, which expresses MYCN under a tetracycline- regulated promoter[31]. DNA immunoprecipitated with an anti-MYCN antibody from untreated or doxycycline-treated (for 2 or 24 h) cells was PCR-amplified using five different pairs of oligonucleotide primers encompassing the five putative MYCN-binding sites (Figure 1C). MYCN was associated with all the putative binding sites; furthermore, the signal decreased in parallel with MYCN downmodulation by doxycycline treatment. These results demonstrate the *in vivo* binding of MYCN with all these sites (Figure 1D).

Finally, we evaluated the effect of MYCN on miRNA *17-5p-92* cluster expression. A ∼3700 bp DNA fragment containing the four upstream MYCN binding sites was cloned at the 5′ site of the luciferase gene in the reporter pGL4 vector (pGL4prom17M) (Figure 1C). Co-transfection of this construct together with an expression vector for MYCN in SH-EP cells led to a sharp transactivation of the luciferase activity, which was not detected in the control empty vector group (Figure 1E). Moreover, in Tet-21/N cells downmodulation of MYCN by doxycycline treatment caused a marked decrease of all miRNAs encoded by the miRNA *17-5p-92* cluster, as evaluated by both Northern blot and qRT-PCR (Figure 1F and data not shown).

Altogether these results indicate that, in neuroblastoma cells, MYCN transcriptionally induces the expression of the miRNA *17-5p-92* cluster by directly binding to its promoter.

### miRNA *17-5p-92* cluster enhances the *in vitro* and *in vivo* tumorigenesis of SK-N-AS neuroblastoma cell line

In order to investigate the oncogenic role of the miRNA *17-5p-92* cluster in neuroblastoma, we generated a stable SK-N-AS transfectant expressing the cluster under a CMV promoter (SK-N-AS *17-5p* cluster). These cells showed an increased expression of the cluster (data not shown), similar to that observed in *MYCN*-amplified neuroblastoma cells (Figure 1B).

**Figure 2:**
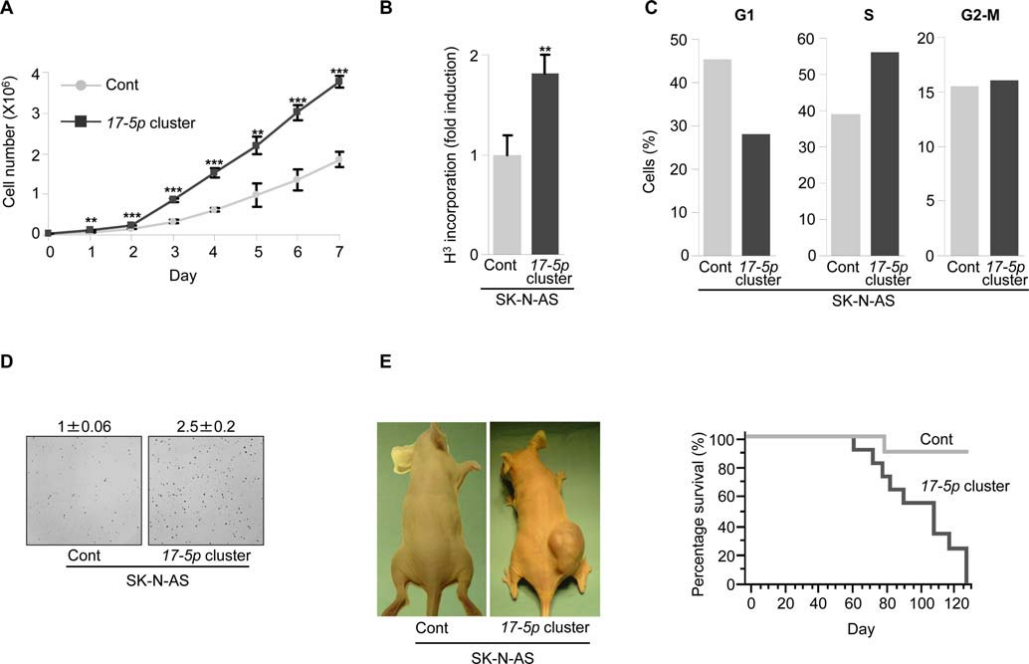
Overexpression of miRNA *17-5p-92* cluster augments *in vitro* and *in vivo* tumorigenesis of SK-N-AS cells. (A) Proliferation curve of SK-N-AS stably transfected with the empty vector (Cont) or the vector expressing the miRNA *17-5p-92* cluster (*17-5p cluster*). Mean±s.d. (*n = 3*). (B) Thymidine incorporation in SK-N-AS Cont or *17-5p* cluster cells. Mean±s.d. (*n = 3*). (C) Cell cycle analysis of SK-N-AS Cont or *17-5p* cluster cells. After 30 h starvation, cells were incubated with a complete medium for 16 h before BrdU incorporation and FACS analysis. Percentage of cells in G1, S or G2-M phase of the cell cycle is indicated. A representative experiment is shown. (D) Anchorage independent growth of SK-N-AS Cont or *17-5p* cluster cells. Cells were plated in a soft agar semisolid medium and colonies were counted after 2 weeks. In each experiment, cells were plated in triplicate. Representative fields are shown. Numbers indicate the fold increase of the colony number formed by SK-N-AS *17-5p* cluster cells relative to SK-N-AS Cont cells (set as 1). Mean±s.d. (*n = 5*); P<0.001. (E) *Left panel*. Nude mice were injected with SK-N-AS Cont or *17-5p* cluster cells and photographed four months after the injection. Representative mice are shown. *Right panel*. Kaplan-Meier curves showing survival of mice injected with SK-N-AS Cont (grey line) or *17-5p* cluster cells (black line); P<0.05 (*n = 11*). ** P<0.01, *** P<0.001.

In a series of *in vitro* experiments, ectopic expression of the cluster induced: (i) an increase of the proliferation rate , as compared to control cells stably transfected with an empty vector (SK-N-AS Cont) (Figure 2A, B); (ii) a decline of the percentage of cells in G1 phase and an inverse rise of the S phase population, demonstrating an accelerated cell cycle progression (Figure 2C); (iii) an increase of the number of colonies formed in a soft-agar semisolid medium, demonstrating a rise of the tumorigenic ability (Figure 2D).

To evaluate the effect of the cluster on *in vivo* tumorigenesis, we injected SK-N-AS Cont or SK-N-AS *17-5p* cluster cells into nude mice. All mice (11/11) injected with SK-N-AS *17-5p* cluster cells developed a tumor and died within 130 days from injection, whereas only 36% of mice (4/11) injected with SK-N-AS Cont cells showed a visible tumor (Figure 2E).

Altogether, these data demonstrate that the miRNA *17-5p-92* cluster enhances cell proliferation and promotes tumorigenesis, both *in vitro* and *in vivo*.

### miR-*17-5p* is responsible for the tumorigenic effect of the miRNA *17-5p-92* cluster through direct downmodulation of p21

To identify potential target genes of the miRNAs encoded by the miRNA *17-5p-92* cluster, we used two algorithms, Target Scan[32] and PicTar[33]. Both indicated that p21 is a candidate target of miR-*17-5p* and -*20a*. Since miR-*17-5p* and -*20a* share similar sequences and functions[7], [14], we focused on miR-*17-5p* for further analysis of p21 targeting.

**Figure 3:**
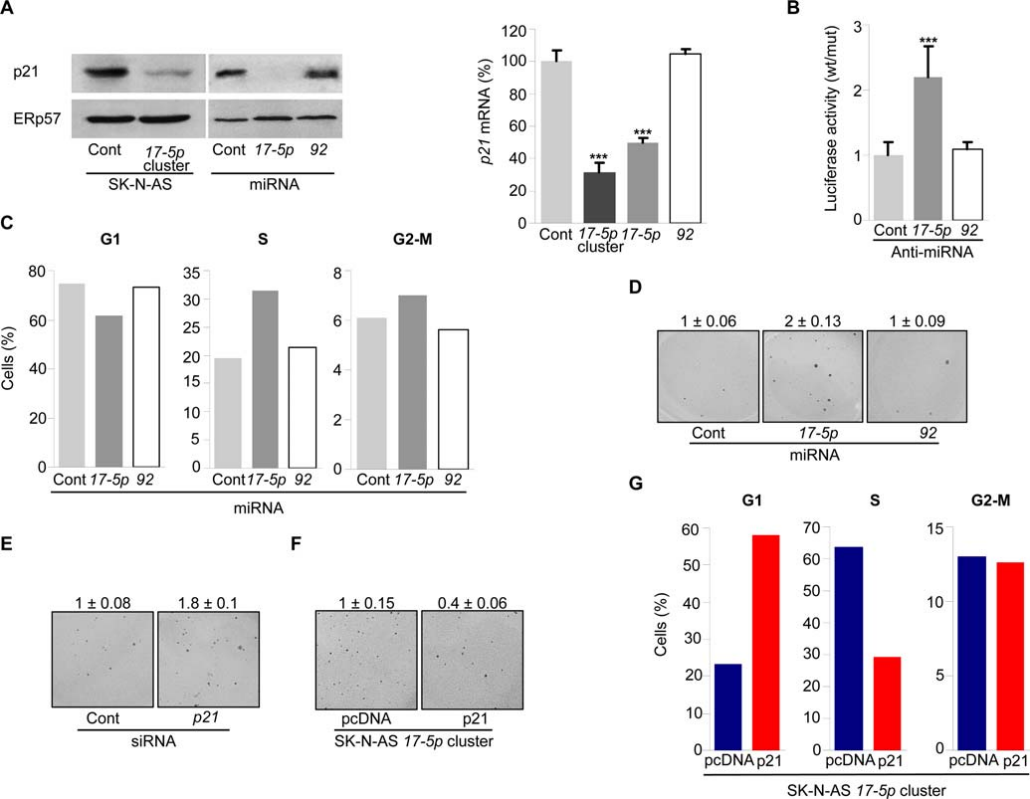
miR-*17-5p* mediates the oncogenic function of miRNA *17-5p-92* cluster through p21 and cell cycle regulation. (A) Western blot (*left panel*) and qRT-PCR (*right panel*) of p21 expression in SK-N-AS Cont or *17-5p* cluster cells, or in SK-N-AS transiently transfected with miR-*17-5p*, -*92* or a control miRNA. A representative Western blot is shown. Mean±s.d. (*n = 3*). (B) Luciferase activity in Tet-21/N cells transfected with pGL3-prom-p21UTR wt or mut in combination with a control or anti-miRNA oligonucleotides complementary to miR-*17-5p* or -*92*. The ratio of normalized luciferase activity in pGL3-prom-p21UTR wt versus mut transfected cells is indicated. Mean±s.d. (*n = 6*). (C) Cell cycle analysis of SK-N-AS cells transfected with miR-*17-5p*, -*92* or a control miRNA. After transfection, cells were starved for 30 h and then incubated with a complete medium for 16 h before BrdU incorporation and FACS analysis. Percentage of cells in G1, S or G2-M phase of the cell cycle is indicated. A representative experiment is shown. (D) Anchorage independent growth of SK-N-AS cells transfected with miRNA-*17-5p*, -*92* or a control miRNA. Cells were plated in a soft agar semisolid medium and colonies were counted after 2 weeks. In each experiment, cells were plated in triplicate. A representative field is shown. Numbers indicate the fold increase of the colony number formed by SK-N-AS transfected with miR-*17-5p* or -*92* relative to SK-N-AS cells transfected with a control miRNA (set as 1). Mean±s.d. (*n = 3*); P<0.001. (E) Colony formation of SK-N-AS cells transfected with siRNA targeting *p21* mRNA (*p21* siRNA) or a control oligonucleotide (Cont siRNA). After transfection, cells were plated in a soft agar semisolid medium and colonies were counted after 2 weeks. In each experiment, cells were plated in triplicate. A representative field is shown. Numbers indicate the fold increase of the colony number formed by SK-N-AS transfected with *p21* siRNA relative to SK-N-AS cells transfected with a control siRNA (set as 1). Mean±s.d. (*n = 3*); P<0.001. (F) Colony formation of SK-N-AS *17-5p* cluster cells stably transfected with an expression vector for p21 or the empty plasmid (pcDNA). Cells were plated in a soft agar semisolid medium and colonies were counted after 2 weeks. In each experiment, cells were plated in triplicate. A representative field is shown. Numbers indicate the fold increase of the colony number formed by SK-N-AS *17-5p* cluster cells transfected with an expression vector for p21 relative to cells transfected with the empty plasmid (set as 1). Mean±s.d. (*n = 3*); P<0.001. (G) Cell cycle analysis of SK-N-AS *17-5p* cluster cells stably transfected with an expression vector for p21 or the empty plasmid (pcDNA). Cells were starved for 24 h and then incubated with a complete medium for 16 h before BrdU incorporation and FACS analysis. Percentage of cells in G1, S or G2-M phase of the cell cycle is indicated. A representative experiment is shown. *** P<0.001.

We first evaluated the expression of p21 in Tet-21/N cells treated or not with doxycycline for 96 h. Downmodulation of MYCN and miR-*17-5p* caused a strong increase of p21 expression at both mRNA and protein level (Figures 1F and S1). Consistently, we observed a clear reduction in the level of endogenous p21 mRNA and protein in SK-N-AS *17-5p* cluster cells, as well as in SK-N-AS transiently transfected with miR-*17-5p*, but not with miR-*92* (Figure 3A).

**Figure 4:**
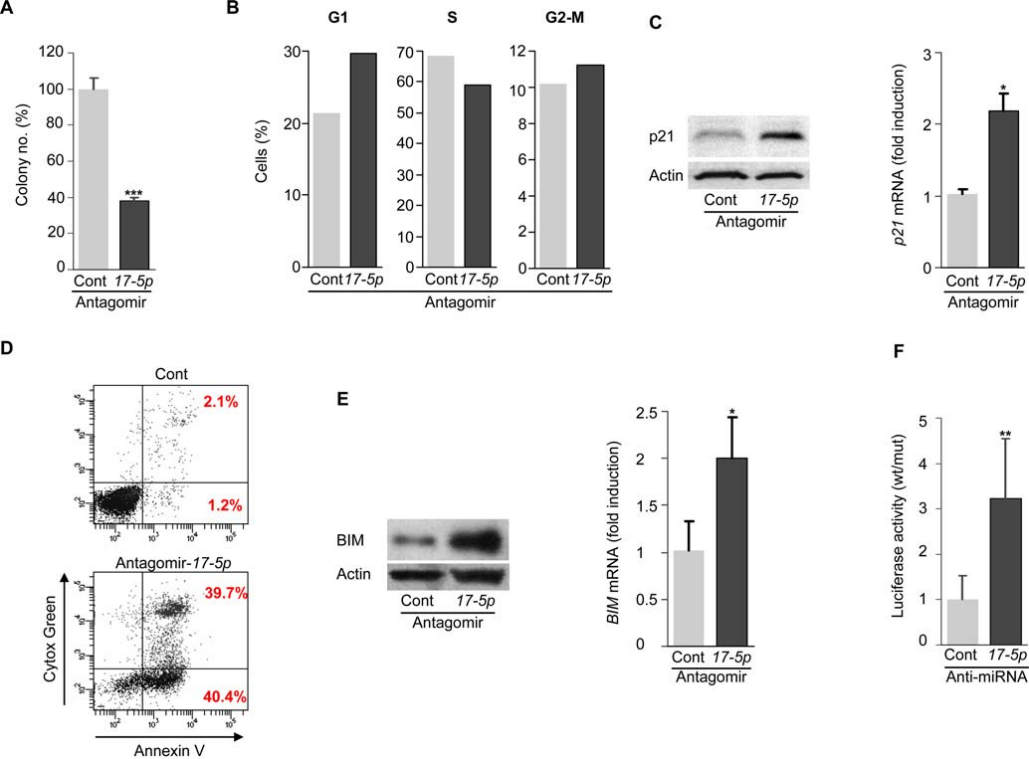
Treatment of *MYCN*-amplified LAN-5 cells with antagomir-*17-5p* inhibits *in vitro* tumorigenesis through p21 and BIM upmodulation. (A) Colony formation of LAN-5 cells treated with antagomir-*17-5p* or a control antagomir. 24 h after treatment, cells were plated in a soft agar semisolid medium and colonies were counted after 2 weeks. In each experiment, cells were plated in triplicate. Mean±s.d. (*n = 3*). (B) Cell cycle analysis of LAN-5 cells treated with antagomir-*17-5p* or a control antagomir. After treatment, cells were starved for 24 h and then incubated with a complete medium for 16 h before BrdU incorporation and FACS analysis. Percentage of cells in G1, S or G2-M phase of the cell cycle is indicated. A representative experiment is shown. (C) Western blot (*left panel*) and qRT-PCR (*right panel*) of p21 expression in LAN-5 cells treated with antagomir-*17-5p* or a control antagomir. A representative Western blot is shown. Mean±s.d. (*n = 3*). (D) Apoptosis of LAN-5 cells treated with antagomir-*17-5p* or a control antagomir. 24 h after treatment, cells were incubated with Annexin V and Cytox Green and analyzed by FACS. A representative experiment is shown. (E) Western blot (*left panel*) and qRT-PCR (*right panel*) of BIM expression in LAN-5 cells treated with antagomir-*17-5p* or a control antagomir. A representative Western blot is shown. Mean±s.d. (*n = 3*). (F) Luciferase activity in Tet-21/N cells transfected with pGL3-prom-BIMUTR wt or mut in combination with a control or an anti-miRNA oligonucleotide complementary to miR-*17-5p*. The ratio of normalized luciferase activity in pGL3-prom-BIMUTR wt versus mut transfected cells is indicated. Mean±s.d. (*n = 6*). * P<0.05, ** P<0.01, *** P<0.001.

To demonstrate that miR-*17-5p* directly regulates p21 expression by binding to its 3′ UTR, we cloned the p21 3′ UTR into the pGL3-Promoter vector, downstream the luciferase gene (pGL3-Prom-p21UTR-wt). As a control, we cloned a mutated p21 3′ UTR, containing 4 mutations in the miR-*17-5p* binding site (pGL3-Prom-p21UTR-mut). Transfection of pGL3-Prom-p21UTR-wt together with an anti-miR-*17-5p* oligonucleotide, but not with either a scrambled anti-miR-*17-5p* or an anti-miR-*92*, led to an increase of the luciferase activity in Tet-21/N cells, due to inhibition of the endogenous miR-*17-5p*. Conversely, the anti-miR-*17-5p* oligonucleotide did not increase the luciferase activity of pGL3-Prom-p21UTR-mut, thus demonstrating that mutation of the miR-*17-5p* binding site in the p21 3′ UTR abolished the ability of miR-*17-5p* to regulate its expression (Figure 4B). Transfection of anti-miR-*20a* enhanced the luciferase activity of PGL3-Prom-p21UTR-wt, as observed for anti-miR-*17-5p*, thus suggesting that miR-*20a* also targets *p21* mRNA (data not shown).

SK-N-AS transfected with miR-*17-5p* showed a higher percentage of cells in the S phase and a lower number of cells in G1 phase, similarly to SK-N-AS *17-5p* cluster cells, whereas miR-*92* did not affect cell cycle progression (Figures 2C and 3C). Since regulation of p21 expression by miRNA *17-5p-92* cluster is essentially mediated by miR-*17-5p*, we hypothesized that the effects of the cluster on the *in vitro* tumorigenesis of SK-N-AS cells were also mediated by miR-*17-5p*. In fact, overexpression of miR-*17-5p*, but not of miR-*92*, increased the number of colonies formed by SK-N-AS in a semisolid medium, as observed for cells overexpressing the entire cluster (Figures 2D and 3D). Altogether, these results show that miR-*17-5p* is the major effector of MYCN-mediated *in vitro* tumorigenesis of SK-N-AS cells.

The role of p21 in the control of cell cycle progression and tumorigenesis of SK-N-AS cells was demonstrated by knocking down p21 with siRNA. Notably, silencing of p21 was associated with an accelerated cell cycle progression, as well as an increased ability of these cells to form colonies in a semisolid medium, as observed upon overexpression of either the miRNA *17-5p-92* cluster or miR-*17-5p* (Figure 3E and data not shown). Importantly, restoration of p21 in SK-N-AS *17-5p* cluster cells abolished the *in vitro* tumorigenic activity of these cells by blocking miRNA *17-5p-92* cluster-induced cell cycle acceleration (Figure 3F, G). Finally, overexpression of miR-*17-5p* in SH-EP cells (a *MYCN*-not-amplified neuroblastoma cell line) enhanced cell proliferation through downmodulation of p21, as observed in SK-N-AS cells, thus demonstrating that these effects were not restricted to a particular cell line (data not shown).

Altogether, these results show that downregulation of p21 mediates miR-*17-5p* induced tumorigenesis in neuroblastoma cell lines.

### Knockdown of miR-*17-5p* decreases the *in vitro* tumorigenesis of *MYCN*-amplified neuroblastoma cells

To determine whether miR-*17-5p* mediates tumorigenesis in *MYCN*-amplified neuroblastoma cells, we evaluated the effect of miR-*17-5p* knockdown in LAN-5 cell line, which expresses miR-*17-5p* at elevated level (Figure 1B). In preliminary experiments, treatment of LAN-5 with antagomir-*17-5p* (a chemically modified anti-miR-*17-5p* oligonucleotide conjugated with cholesterol[34]) efficiently downmodulated miR-*17-5p* expression, as compared to cells treated with PBS or control antagomir-*1* (data not shown). Incubation of LAN-5 with antagomir-*17-5p*, but not with the control antagomir, markedly inhibited cell proliferation, decreased *in vitro* tumorigenesis in soft agar and blocked cell cycle progression (Figure 4A, B and data not shown). Furthermore, inhibition of miR-*17-5p* was associated with an increase of p21 mRNA and protein level (Figure 4C).

In addition, knockdown of miR-*17-5p* strongly promoted both early and late apoptosis (Figure 4D). Notably, p21 overexpression did not induce apoptosis in LAN-5 (data not shown), thus implying that an additional miR-*17-5p* target may regulate this process. Bioinformatic analysis with Target Scan[32], Pic Tar[33] and miRanda[35] indicated the pro-apoptotic factor BIM as a putative target of miR-*17-5p*. Consistently, knockdown of miR-*17-5p* by antagomir increased BIM expression at both mRNA and protein level in LAN-5 cells (Figure 4E). To demonstrate that this regulation occurs through a direct binding of miR-*17-5p* to BIM 3′ UTR, we cloned a portion of the BIM 3′ UTR containing the miR-*17-5p* putative binding site into the pGL3-Promoter vector, downstream the luciferase gene (pGL3-Prom-BIMUTR-wt). As a control, we cloned a region of BIM 3′ UTR containing a mutated miR-*17-5p* recognition site (pGL3-Cont-BIM UTR-mut). Co-transfection of pGL3-Prom-BIMUTR-wt together with an anti miR-*17-5p*, but not with a scrambled anti-miR-*17-5p* oligonucleotide, caused an increase of the luciferase activity in Tet-21/N cells (Figure 4F). This increase was not observed in cells transfected with the pGL3-Cont-BIM UTR-mut construct, thus demonstrating that mutation of the miR-*17-5p* binding site in the BIM 3′ UTR abolishes the ability of miR-*17-5p* to regulate its expression (Figure 4F).

### Knockdown of miR-*17-5p* inhibits the *in vivo* tumorigenic ability of *MYCN*-amplified neuroblastoma cells

**Figure 5:**
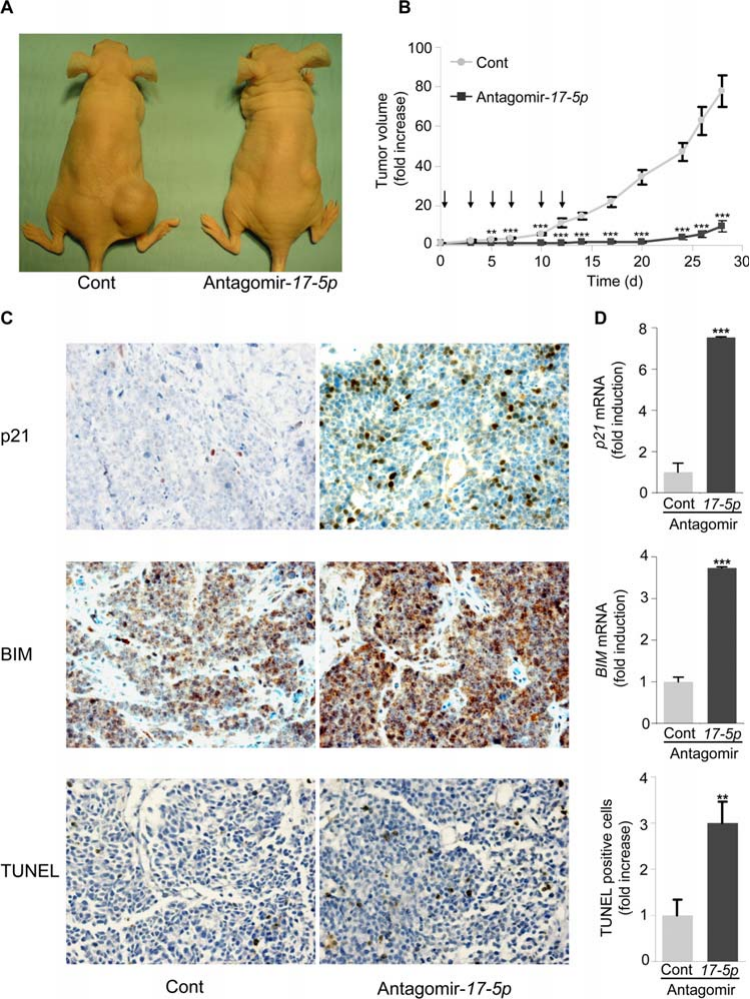
Treatment of *MYCN*-amplified LAN5 cells with antagomir-*17-5p* inhibits *in vivo* tumorigenesis through p21 and BIM upmodulation. (A) Nude mice were injected with *MYCN*-amplified LAN-5 cells and treated when tumors reached 150 mm^3^ with antagomir-*17-5p* or a control antagomir for 2 weeks. Representative mice were photographed four weeks after the first injection. (B) Growth curves of LAN-5 tumors treated with antagomir-*17-5p* or a control antagomir for 2 weeks (injections of antagomirs are indicated by arrows). The volume of the tumors was measured three times weekly and is plotted as the fold increase relative to the day of the first antagomir injection (day 0), set as 1. Mean±s.e.m. (*n = 11* for cont; *n = 10* for Antagomir-*17-5p*). (C) Immunohistochemistry on LAN-5 tumors treated with antagomir-*17-5p* or a control antagomir for 24 h. Sections derived from tumors were incubated with an anti-p21 antibody (*upper panels*), an anti-BIM antibody (*middle panels*) or with TUNEL for detection of apoptotic cells (*lower panels*). Representative fields are shown. (D) qRT-PCR of *p21* (*upper panel*) or *BIM* (*middle panel*) mRNA levels in tumors formed by LAN-5 cells and treated with antagomir-*17-5p* or a control antagomir for 24 h. Mean±s.d. (*n = 3*). *Lower panel*. TUNEL positive cells in tumors treated with antagomir-*17-5p* or a control antagomir for 24 h. Mean±s.e.m. (*n = 16*). ** P<0.01, *** P<0.001.

Based on the *in vitro* studies, we hypothesized that abolition of miR-*17-5p* expression may inhibit tumor growth *in vivo*. To address this critical question, *MYCN*-amplified LAN-5 cells were injected into nude mice, and tumors thereby generated were treated with antagomir-*17-5p* or a control antagomir for two weeks. Injection of antagomir-*17-5p* dramatically inhibited tumor growth: this effect , already relevant after one week of therapy was maintained through the end of the treatment, leading in 30% of cases to complete regression of the tumor mass (Figure 5A, B). Conversely, administration of the control antagomir did not affect tumor development, as observed in PBS-treated tumors (data not shown). Tumor analysis at 24 h after the first administration of antagomir-*17-5p* showed a marked downmodulation of miR-*17-5p*, associated with a strong increase of p21 and BIM at both mRNA and protein level (Figure 5C, D and data not shown). Consistently, TUNEL assay showed an increased apoptosis in tumors treated with antagomir-*17-5p*, as compared to the control group (Figure 5C, D).

Altogether, these results demonstrate that *in vivo* treatment of *MYCN*-amplified neuroblastoma with antagomir-*17-5p* abolishes tumor growth by upmodulation of p21 and BIM and increased apoptosis.

### Expression of MYCN, miR-*17-5p* and *p21* in primary neuroblastoma samples

**Figure 6:**
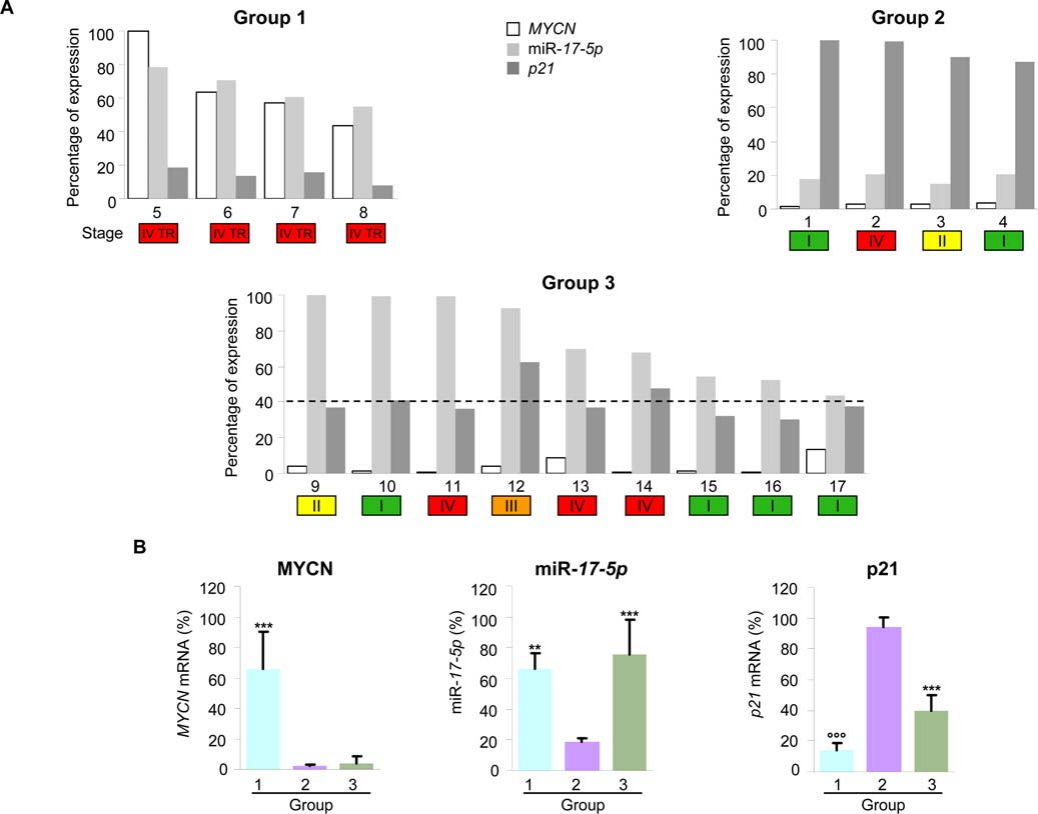
miR-*17-5p* expression is correlated to *MYCN* and *p21* levels in human primary neuroblastomas. (A) Expression of *MYCN*, miR-*17-5p* and *p21* was analyzed in primary tumors by qRT-PCR. Depending on the expression levels of *MYCN* and miR-*17-5p*, primary tumors were divided into three groups. Group 1 (*left upper panel*) includes *MYCN*-amplified tumors displaying high levels of miR-*17-5p* and very low levels of *p21*. Group 2 (*right upper panel*) includes *MYCN*-not-amplified tumors expressing low levels of miR-*17-5p* and high levels of *p21*. Group 3 (*lower panel*) includes *MYCN*-not-amplified tumors expressing high levels of miR-*17-5p* and moderately low levels of *p21* (a 40% decrease baseline is shown). The coloured boxes indicate the tumor stage (from I to IV) and the therapy resistant tumors (TR). (B) Expression of *MYCN*, miR-*17-5p* and *p21* in the three groups of primary tumors. Mean±s.d.. ** P<0.01, *** P<0.001 when compared to second group; °°° P<0.001 when compared to the other groups.

Finally, we evaluated the expression of *MYCN*, miR-*17-5p* and *p21* mRNA in freshly dissected primary neuroblastomas, including *MYCN*-amplified and not-amplified tumors (Figure 6). In *MYCN*-amplified tumors the upmodulation of *MYCN* was associated with elevated miR-*17*-5p expression and markedly low levels of *p21* (group 1). *MYCN*-not-amplified neuroblastomas, showing a low level of *MYCN*, displayed two distinct patterns: (i) 30% of cases were characterized by low miR-*17-5p* expression and high-level of p21 (group 2); (ii) 70% of samples unexpectedly showed an elevated expression of miR-*17-5p*, associated with moderate downmodulation of *p21* (group 3), less pronounced than in group 1.

## Discussion

Neuroblastoma is one of the most common extra-cranial solid tumor of early childhood, accounting for >15% of cancer-related deaths in children. While the clinical diversity of neuroblastoma correlates with several genetic features, such as ploidy or allelic loss, the amplification of the *MYCN* gene is the best genetic marker of poor prognosis[36]. However, the mechanisms underlying MYCN-mediated neuroblastoma progression have not been identified. Our work describes a novel oncogenic pathway underlying neuroblastoma development, whereby MYCN transactivates the miRNA *17-5p-92* cluster, which in turn downmodulates the tumor suppressors p21 and BIM. Among the different miRNAs pertaining to the miRNA *17-5p-92* cluster, miR-*17-5p* and miR-*20a*, which show an almost complete homology, are the only ones targeting p21, as indicated by bioinformatic analysis and luciferase assay. Furthermore, miR-*17-5p* level in neuroblastoma cell lines is often more elevated than that of miR-*20a*. Therefore, we focused on miR-*17-5p* as the primary effector of MYCN-mediated tumorigenesis.

Our studies indicate that, in neuroblastoma cell lines, miR-*17-5p* controls cell cycle progression through p21. In diverse tumors miR-*106b*, structurally related to but functionally distinct from miR-*17-5p* [7], regulates cell cycle progression through p21[37]. However, we observed that miR-*106b* is not upmodulated in *MYCN*-amplified neuroblastoma cells (data not shown), thus suggesting that in neuroblastoma miR-*106b* is not involved in p21 regulation.

p21 is a tumor suppressor gene, whose expression is mainly regulated at transcriptional level by p53[38]. Although p53 is the most frequently mutated gene in human cancers, p53 mutations have not been detected in primary neuroblastoma[39]. In addition, lack of correlation between p53 and p21 levels in *MYCN*-amplified neuroblastoma cell lines[40] suggests that expression of p21 may be p53-independent in neuroblastoma. Our study shows a novel p53-independent mechanism for the regulation of p21 expression. In fact, we report that miR-*17-5p* directly regulates p21 in both the p53 knockout SK-N-AS cell line and the SH-EP and LAN-5 cells expressing endogenous p53[41]. Furthermore, treatment of LAN-5 with antagomir-*17-5p* caused an upmodulation of p21 without affecting p53 levels (data not shown). Id2 has also been proposed to mediate MYCN ability to bypass the cell cycle checkpoint imposed by Rb[42]. However, a direct binding of MYCN to Id2 promoter has not been demonstrated and Id2 expression does not seem to be associated with MYCN amplification or expression in human neuroblastoma[43].

Treatment of LAN-5 with antagomir-*17-5p* causes not only a block of cell cycle, but also a dramatic apoptosis. Tumor progression usually occurs through activation of different pathways, leading to increased cell proliferation and protection from apoptosis, which provides a survival advantage to cancer cells. Oncoproteins of the myc family promote apoptosis[44]–[46]. However, *MYCN* amplification in neuroblastoma causes resistance to chemotherapy, associated with tumor progression and poor prognosis[36]: this suggests that MYCN-induced apoptosis may be inhibited by an additional oncogenic mechanism, crucial for tumor progression. Our findings indicate that miR-*17-5p* is a key factor inducing protection from MYCN-primed apoptosis in neuroblastoma. Indeed, knock down of miR-*17-5p* is sufficient to promote massive apoptosis of *MYCN*-amplified LAN-5 cells. This occurs through upmodulation of the proapoptotic factor BIM, mediated by direct binding of miR-*17-5p* to *BIM* mRNA 3′ UTR.

**Figure 7:**
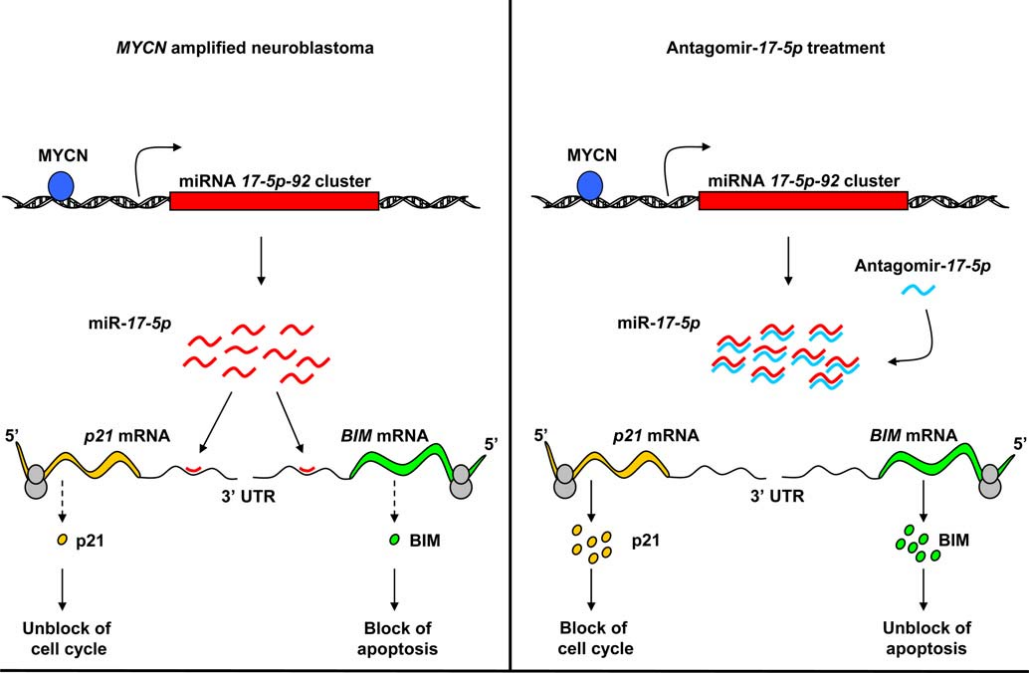
Mechanism of antagomir-*17-5p* action in the treatment of *MYCN*-amplified neuroblastoma. In *MYCN*-amplified neuroblatoma, MYCN binds the miRNA *17-5p-92* cluster promoter and transactivates the miR-*17-5p*. High levels of miR-*17-5p* inhibit the translation of *p21* and *BIM* through the direct binding to their mRNA 3′ UTRs. Low levels of p21 and BIM lead to an accelerated cell cycle progression and a resistance to apoptosis, respectively. Treatment of *MYCN*-amplified cells with antagomir-*17-5p* inhibits miR-*17-5p* function, thus allowing the translation of both *p21* and *BIM* mRNA. High levels of p21 and BIM cause a block of cell cycle and induction of apoptosis, respectively, thus inhibiting tumor growth.

The “combinatorial circuitry model”, predicts that a single miRNA may target multiple mRNAs. In *MYCN*-amplified neuroblastoma MYCN transactivates miR-*17-5p*, which in turn accelerates cell cycle progression by downmodulating p21 and protects cells from apoptosis by inhibiting BIM expression (Figure 7). This occurs in a p53-independent manner, thus providing a mechanistic explanation for the rarity of p53 mutations in neuroblastoma.

A second set of studies was focused on the molecular analysis of primary neuroblastoma samples, derived from a well characterized series of patients. The samples were divided in three groups. The first and second ones yielded expected results. Specifically, *the first group* is characterized by *MYCN*-amplification, poor prognosis and therapy resistance, coupled with a marked rise of miR-*17-5p* level and a dramatic decrease of *p21*. *The second one* features low levels of *MYCN* and miR-*17-5p*, associated with elevated *p21* expression: these patients show a relatively benign clinical profile, characterized by slow disease progression and therapy response. Surprisingly, *the third*, *large group* of samples shows an elevated level of miR-*17-5p* in the absence of increased *MYCN* expression. The levels of *p21* are intermediate between those of the first and the second groups, while the clinical features are relatively benign. In line with these clinical observations, we observed that, in neuroblastoma cell lines with normal MYCN level, overexpression of miRNA *17-5p-92* cluster, as well as miR-*17-5p* alone, is able to enhance tumorigenesis. It is apparent, therefore, that miR-*17-5p* does not require MYCN to exert its oncogenic activity in neuroblastoma. Notably, knock down of miR-*17-5p* by antagomir sharply inhibits the *in vitro* and *in vivo* tumorigenesis of *MYCN*-amplified LAN-5 cells, suggesting that miR-*17-5p* is a key oncogenic factor in both *MYCN*-amplified and not-amplified neuroblastoma.

The lower level of p21 observed in the first group, as compared to the third one, may be due to a direct downmodulation of p21 by MYCN. In fact, c-Myc negatively regulates the expression of p21 at transcriptional level[47]. Preliminary results indicate that MYCN binds the p21 promoter, suggesting that in *MYCN*-amplified neuroblastoma MYCN exerts an additional suppressing activity on p21 expression at transcriptional level (data not shown). In the clinical setting, the remarkably low level of p21 in the second group is probably linked to its poor prognosis and therapy resistance.

Our antagomir studies bear a potential significance at clinical level. Despite recent advances in treatment options, aggressive neuroblastoma carrying *MYCN* amplification is refractory to current therapy, leading to a disease related mortality of up to 70%. Therefore, the development of new therapeutic approaches is warranted. Strategies based on *MYCN* repression by siRNA may prove unsatisfactory: in fact, efficient inhibition of MYCN levels is hampered by the high levels of MYCN. Conversely, antagomir-*17-5p* treatment may be beneficial in *MYCN*-amplified neuroblastoma, also in view of evidence suggesting that systemic antagomir treatment is not coupled with significant toxicity[34], [48]. In conclusion, our results provide the first demonstration that antagomirs can efficiently inhibit tumor growth *in vivo*, thus raising the possibility that these molecules may ultimately be clinically useful in the treatment of cancer.

## Materials and Methods

### Cell culture and tumor samples

The SH-EP and SK-N-AS human neuroblastoma cell lines express low levels of MYCN whereas SH-SY-5Y, LAN-5 and IMR32 overexpress MYCN (LAN-5 and IMR32 as a result of gene amplification). All the cells, obtained from the American Type Culture Collection, (Manassas, VA), were grown in RPMI medium supplemented with 10% FBS (HyClone, Logan, Utah). Tumor samples were obtained from patients diagnosed with neuroblastoma after informed consent of their parents admitted to the Division of Oncology at Bambino Gesù Children's Hospital. Samples were freshly resected during surgery and immediately frozen in liquid nitrogen for subsequent total RNA extraction. Tumors were classified according to the International Neuroblastoma Pathology Classification (INCP): 6 were at stage I, 2 at stage II, 1 at stage III, and 8 at stage IV.

For proliferation assay, cells were seeded at the same density (2.5×10^4^ cells/ml) and counted at the indicated times. For thymidine incorporation assay, 50×10^3^ cells/well were plated in 96 well plates in triplicated; 24 h after seeding, each well was incubated with 1 µCi of [^3^H] thymidine (Amersham Biosciences). After 16 h, the cells were harvested and analysed by liquid scintillation in a Microcounter (Wallac). The counts from triplicate wells were averages.

The *in vitro* tumorigenesis of neuroblastoma cell lines was determined by seeding the cells at low density (400–600 cells/ml) in 1.5 ml of 0.3% Agar Noble (Difco, Kansas City, Missouri) and RPMI-10% FBS and plating them on 1.5 ml of 0.6% Agar Noble and RPMI-10% FBS. Colony formation was determined after two weeks by staining with crystal violet (Fluka, St. Gallen, Switzerland) and colonies were counted visually.

SK-N-AS Cont and SK-N-AS *17-5p* cluster cells were obtained by transfection of the SK-N-AS cells with the empty vector or the miRNA *17-5p-92* cluster expression constructs, followed by two weeks of blasticidin selection at 0.5 µg/ml. SK-N-AS *17-5p* cluster cells stably expressing the p21 expression vector or the empty pcDNA3, were obtained by two weeks of hygromicin selection at 100 µg/ml, after transfection with Lipofectamine 2000 (Invitrogen, Carlsbad, CA).

### Oligonucleotides and transfection experiments

For transfection experiments, cells were seeded in antibiotic-free media for 24 h and then transfected with Lipofectamine 2000 according to manufacturer's instructions (Invitrogen). Transfection of a pool of four siRNA oligonucleotides specifically targeting *p21* (Smart Pool siRNA, Dharmacon, Lafayette, CO), at the final concentration of 10 nM, was performed with Hiperfect (Qiagen, Hilden, Germany). When indicated cells were serum starved for 30 h before transfection.

In promoter assays, SH-EP cells were transfected with 0.6 µg of firefly luciferase vectors (empty pGL4 or pGL4prom17M vector), in combination with 1.8 µg of pcDNA3 (Promega corporation, Madison, WI) or pIRV neo SV-MycN, together with a *Renilla* luciferase vector (50 ng) as internal control.

In luciferase experiments, Tet-21/N cells were transfected with 0.4 µg of firefly luciferase vectors (empty pGL3-prom, pGL3-prom-p21UTR wt or mutant, pGL3-prom-BIM UTR wt or mutant) and 50 ng of *Renilla* luciferase vector, together with 160 nM *2*′-*O*-Methyl oligonucleotides (anti-*17-5p* 5′-ACUACCUGCACUGUAAGCACUUUG-3′; anti-*92* 5′-CAGGCCGGGACAAGU GCAAUA-3′; anti-control 5′-UUCGACGCGGAAUACUUCGAU-3′; Dharmacon), where indicated. Firefly luciferase experiments were measured 48 or 72 h after transfection by using Microlite TLX1 (Dynatech Laboratoires, Chantilly, CA) and then normalized for *Renilla* luciferase activity.

Transfection of SK-N-AS was performed with stability enhanced mature miRNA *17-5p* (5′-UACCUGCACUGUAAGCACUUUGGU-3′), *92* (5′-GGCCGGGACAAGUGCAAUAUG-3′) or a control (5′-UACUUGCACUAUACUUGUGACAGU-3′) double-stranded RNA oligonucleotides (Dharmacon) at the final concentration of 160 nM.

### Cell cycle analysis

Cells were seeded in 6 well plates at 40% of confluence and incubated at 37°C for 24 h. Cells were then synchronized by serum depletion for 30 h and pulsed with 10 µM BrdU (Sigma) for 30 min at different times after FCS addition. After BrdU incorporation, cells were harvested and fixed in ice-cold 70% ethanol. DNA was denatured with HCl 2N/Triton 20% and labeled with an anti-BrdU antibody (BD Bioscience) for 1 h. Then, cells were resuspended in washing buffer and labeled with anti-mouse APC-conjugated antibody (Beckton Dickinson). Labeled cells were washed and resuspended in PBS containing 5 µg/ml propidium iodide and analysed on a FACSCanto flow cytometer (Beckton Dickinson) using the DIVA software. All the flow cytometry experiments were performed at least twice and a representative experiment is shown in each figure.

### Tumorigenicity assay in nude mice

Six-week-old nude mice strain C57/BL6 were subcutaneously injected into the right flank with 25×10^6^ cells (SKNAS Cont, SKNAS *17-5p* cluster or LAN-5). Tumor size was assessed every two days by caliper measurement. Tumor volume was calculated as follow: volume = Dxd^2^×π/6, where D and d are the longer and the shorter diameters, respectively. For survival analysis, mice were sacrificed when tumors reached the volume of 500 mm^3^.

### *In vitro* treatment of LAN-5 with antagomir

Antagomirs were synthesized as described[34]. Sequences were 5′-a_s_c_s_uaccugcacuguaagcacu_s_u_s_u_s_g_s_– Chol 3′ (antagomir-*17-5p*), 5′-u_s_a_s_cauacuucuuuacauu_s_c_s_c_s_a_s_- Chol 3′ (control antagomir-1). Lower case letters represent *2*′-*O*-Methyl-modified oligonucleotides, subscript ‘s’ represents a phosphorothioate linkage, and ‘Chol’ represents linked cholesterol.

LAN-5 cells were seeded in antibiotic-free media at 50–60% of confluence (6×10^5^ cells in a six-wells plate) and treated for 24 h with antagomir-*17-5p*, control antagomir at a final concentration of 2.4 µM, or with an equal volume of PBS.Apoptosis was measured with the Apoptosis Detection Kit (MBL International, Woburn, MA) according to manufacturer's instructions. Briefly, 5×10^4^ cells were stained with Annexin V-Cy5 and Cytox Green and analyzed using a BD FACSCanto Cytometer.

### *In vivo* administration of antagomir-*17-5p* in tumors generated by LAN-5 neuroblastoma cells

25×10^6^ LAN5 cells were subcutaneuosly injected into the flank of 6–8-week-old athymic nude mice. After one week, when the tumors reached an average volume of ∼150 mm^3^, the tumor-bearing nude mice were treated with antagomir-*17-5p*. 100 µl of antagomir-*17-5p* (diluted in PBS at 2 mg/ml), or control antagomir, or PBS were injected intratumorally three times per week for two weeks. Tumor diameters were measured at regular intervals as described above.

### DNA constructs

The pGL4prom17M construct was obtained by sequential cloning into the pGL4.10 vector (Promega) of 3 fragments amplified by PCR from human genomic DNA. A 1081 base pair fragment (clone A: nucleotides -3731-2900) was first amplified with the forward *17-92promAfor* (5′-ATAGGTACCCCGGAATTTCCTGAACCACAATG-3′) and the revers *17-92promArev* (5′-GATCTCGAGGGAGTAGCCGCCACCATCTTCGGCT-3′) primers. The obtained DNA was then digested with *KpnI* and *XhoI* and cloned in pGL4.10 vector. A second segment of the cluster promoter (segment B: nucleotides -2649-1425) was obtained with the primers: *17-92promBfor* 5′-GATCTCGAGTCCTGGTGAGTCTGCCCGCCCCT-3′ and *17-92promBrev* 5′-GATAGATCTAACACCCGAGACTGCAAAGTGCCCG-3′ and it was cloned downstream the fragment A by double digestion with *XhoI* and *BglII*. The last portion of the promoter (fragment C: nucleotides -1423-1) was obtained by digestion with *BglII* and *HindIII* of the pGL4prom17 construct[7] and cloned upstream of the luciferase coding sequence.

The p21 3′UTR was PCR amplified from human genomic DNA by using the primers *p21 3′UTRfor* 5′-ATAGCTAGCCACAGGAAGCCTGCAGTCCTGG-3′ and *p21 3′UTRrev* 5′-CCTGCCCTCGAGAGGTTTACAGTCTAGG-3′ and cloned downstream of the luciferase gene in the pGL3-Cont PLK+ vector[7] by digestion with *NheI* and *XhoI* (construct pGL3-Cont-p21UTR-wt). From this construct the pGL3-Cont-p21UTR-mut mutant derivative was generated by inverse PCR with the following primers: *p21 inv for* 5′-AGCAGA AGGGGCCTCACCGAGTGGG-3′ and *p21 inv rev* 5′-CTGGATCTGTTTACTTCTCAAATG-3′.

A 1014-nt-long region of the BIM 3′UTR was obtained by PCR with the forward primer *BIM 3′UTR for* (5′ ATAGGTACCGAAACAGGCCTCATCCCACTTCC-3′) and the reverse primer *BIM 3′UTR rev* (5′-ATAGCTAGCGTTCTGGTGCCAACAGCCTGGTC-3′), containing the *KpnI* and the *NheI* restriction sites, respectively, and cloned downstream of the luciferase stop codon in the pGL3-Cont PLK+ vector. The mutant derivative of this construct (construct pGL3-Cont-BIM UTR-mut) was generated by inverse PCR with the following primers: *BIM inv for* 5′-CCTGTAGA GACTCTGTTTCCTCAGCCCTG-3′ and *BIM inv rev* 5′-TTGAGTCTCACTGGCACGTGAGTA CAGGG-3′.

The pcDNA6.2-GW/EmGFP-miRNA *17-5p* cluster contruct was obtained by cloning a fragment PCR amplified from human genomic DNA into pcDNA6.2-GW/EmGFP-miR vector. The miRNA *17-5p-92* cluster was amplified with the forward primer *17-5p-92*cluster-*BamH1* (5′-GATGGATCCCTAAATGGACCTCATATCTTTGAG-3′) containing the *BamHI* site and the reverse primer *17-5p-92*cluster-*XhoI* (5′-GATCTCGAGGAAAACAAGACAAGATGTATTTAC AC-3′) containing the *XhoI* site, digested with *BamHI* and *XhoI* and cloned in pcDNA6.2-GW/EmGFP-miR vector. pIRV neo SV-MycN and pCEP-WAF1 (p21) were kindly provided by G Giannini and S. Soddu, respectively.

### Northern blot

Total RNA isolation was performed using TRIZOL Reagent (Invitrogen), following the manufacturer's instructions. Northern blot analysis was performed as described[7]. Briefly, RNA samples (30 µg each) were run on 15% acrylamide urea-denaturing precast gel (Invitrogen), and transferred onto Hybond-N+ membrane (Amersham Biosciences, Little Chalfont, UK). The hybridization was performed overnight with DNA probes previously labelled with γ-32P-ATP, at 37 °C in 0.1% SDS with 6× SSC. Membranes were washed at room temperature twice in 0.1% SDS with 2× SSC. The probes used were: miR-*17-5p*, 5′-ACTACCTGCACTGTAAGCACTTTG-3′; miR-*18a*, 5′-TATCTGCACTAGATGCACCTT-3′; miR-*19a*, 5′-TCAGTTTTGCATAGATTT GCACA-3′; miR-*19b*, 5′-TCAGTTTTGCATGGATTTGCACA-3′; miR-*20a*, 5′-CTACCTGCAC TATGAGCACTTTG-3′; miR-*92*, 5′-CAGGCCGGGACAAGTGCAATA-3′; Met-tRNA, 5′-TGGTAGCAGAGGATGGTTTCGATCCATCGACCTCTG-3′.

### Real-time RT–PCR

For mRNA analysis, total RNA was purified with TRIZOL Reagent (Invitrogen). Reverse transcription and Real-time PCR were performed as described[7]. Expression of mature miRNAs was determined using miRNA-specific quantitative real-time PCR (qRT–PCR; Applied Biosystems, Foster City, CA). U6 snRNA was used for normalization.

### Oligonucleotides and transfection experiments

For transfection experiments, cells were seeded in antibiotic-free media for 24 h and then transfected with Lipofectamine 2000 according to the manufacturer's instructions (Invitrogen, Carlsbad, CA). Transfection of a pool of four siRNA oligonucleotides specifically targeting *p21* or *BIM* mRNAs (Smart Pool siRNA, Dharmacon) was performed with Hiperfect (Qiagen, Hilden, Germany).

In promoter assays experiments, SHEP cells were transfected with 0.6 µg of firefly luciferase vectors (empty pGL4 or pGL4prom17M vector), in combination with 1.8 µg of pcDNA3 (Promega corporation, Madison, WI) or pIRV neo SV-MycN, together with a *Renilla* luciferase vector (50 ng) as internal control.

In luciferase experiments, Tet-21/N cells were transfected with 0.4 µg of firefly luciferase vectors (empty pGL3-prom, pGL3-prom-p21UTR wt or mutant, pGL3-prom-BIM UTR wt or del) and 50 ng of *Renilla* luciferase vector, together with 160 nM *2*′-*O*-Methyl oligonucleotides (anti-*17-5p* 5′-ACUACCUGCACUGUAAGCACUUUG-3′; anti-*92* 5′-CAGGCCGGGACAAGUGCA AUA-3′; anti-control 5′-UUCGACGCGGAAUACUUCGAU-3′; Dharmacon, Lafayette, CO), as indicated. Firefly luciferase experiments were measured 48 or 72 h after transfection by using Microlite TLX1 (Dynatech Laboratoires, Chantilly, CA) and then normalized for *Renilla* luciferase activity.

Transfection of SK-N-AS was performed with stability enhanced mature miRNA *17-5p* (5′-UACCUGCACUGUAAGCACUUUGGU-3′), *92* (5′-GGCCGGGACAAGUGCAAUAUG-3′) or a control (5′-UACUUGCACUAUACUUGUGACAGU-3′) double-stranded RNA oligonucleotides (Dharmacon) at the final concentration of 160 nM.

SK-N-AS *17-5p-92* cluster or SKNAS-Cont stable transfectants were selected by adding 0.5 µg/ml blasticidin to the culture medium. Stable p21 transfectants in SK-N-AS-*17-5p-92* were obtained by selection in hygromicin (100 µg/ml).

### Western blot

Whole cell protein extracts (lysis buffer: 50 mM Tris-HCl pH 7.5, 150 mM NaCl, 1% NP40, 1 mM PMSF and 1× Protease Inhibitor Cocktail - Sigma-Aldrich, St.Louis, MO) were quantified by BCA assay (Pierce, Rockford, IL), separated onto NuPAGE 12% polyacrylamide gels (Invitrogen) and blotted on nitrocellulose membranes (Whatman, England). The filters were hybridized with polyclonal anti-MYCN, anti-p21, anti-BIM (Santa Cruz Biotechnology, CA), followed by a secondary anti-rabbit IgG peroxidase-conjugated antibody (Biorad, Hercules, CA). The polyclonal antibody ERp57 and monoclonal antibody anti-actin (Calbiochem, Darmstadt, Germany) were used as loading control. Bands were quantified with Chemi Doc software.

### Chromatin immunoprecipitation

Chromatin immunoprecipitation was performed as described[49] using an anti-MYCN antibody and amplifying the immunoprecipitated DNA by PCR with the following oligonucleotides derived from the genomic region upstream and downstream the microRNA *17-5p-92* cluster: fragments 1 forward 5′-CACAGCGTACACGTCGAGTC-3′ and reverse 5′-CCCCACTCCCTCATTAGCAT-3′; fragments 2 forward 5′-CTGGGGGACACAAAGGAG-3′ and reverse 5′-AACACCCGAGAC TGCAAAGT-3′; fragments 3 forward 5′-TTGCTGTTAGGAGGTTGGAAA-3′ and reverse 5′-CCCATTCCAGAAAACTTCCTT-3′; fragments 4 forward 5′-GGAAGCCAGAAGAGGAGGAA-3′ and reverse 5′-CACTGGAAGTGGTGGCTCTT-3′; fragments 5 forward 5′-CCAGACTTTGGCAACAGTGA-3′ and reverse 5′-TTGCCTGTACTTCAGCTTGG-3′. Control for non-specific DNA immunoprecipitation was produced by amplifying a fragment of the β-*actin* gene with the following oligonucleotides: forward 5′-CCCTCCAAGAGCTCCTTCTG-3′ and reverse 5′-TGTGCTCGCGGGGCGGACGC-3′.

### Immunohistochemistry

Expression of p21 and BIM was analyzed by immunohistochemistry on 5-µm slices formalin-fixed paraffin-embedded sections of tumor xenografts by using monoclonal antibody to p21 (Cell Signaling, Danvers, MA) and polyclonal antibody to BIM (ProSci Incorporated, Poway, CA). Antigen was retrieved by pretreating dewaxed sections in a microwave oven at 750 W for 5 minutes in citrate buffer (pH 6) and processing them with a Super Sensitive Link-Labeled Detection System (Biogenex, Menarini, Florence, Italy). The enzymatic activity was developed using 3-amino-9-ethylcarbazole (AEC, Dako, Milan, Italy) as a chromogenic substrate. Following counterstaining with Mayer's haematoxylin, slides were mounted in aqueous mounting medium (glycergel, Dako).

*In situ* detection of apoptosis in formalin-fixed, paraffin-embedded tumor tissues was performed by TUNEL assay (Apoptag Plus, Chemicon, Prodotti Gianni), as previously reported[50].

### Statistical analysis

Data are presented as mean and error bars indicate the standard deviation (s.d.) or the standard error (s.e.m.). The groups were compared by one-way analysis of variance (Anova, Chicago, IL) using Bonferroni's test or by the unpaired t-test with two-tailed p value. Survival data are presented as Kaplan-Meyer plots and were analysed using a log-rank (Mantel-Haenszel) method. Significance level was P<0.05.

## Supporting Information

Figure S1 p21 is upmodulated in Tet-21/N cells upon treatment with doxycyclin Western blot (left panel) and qRT-PCR (right panel) of p21 expression in Tet-21/N untreated or treated with doxycyclin for 96 h. A representative Western blot is shown. Mean±s.d. (n = 3). *** P<0.001.
(0.23 MB PPT)
