# Supplementary figures and images for: Antagomir-17-5p Abolishes the Growth of Therapy-Resistant Neuroblastoma through p21 and BIM
Source: PLoS One. 2008 May 21;3(5):e2236. doi: 10.1371/journal.pone.0002236 (PMC2375057; doi:10.1371/journal.pone.0002236)

## Slide 1
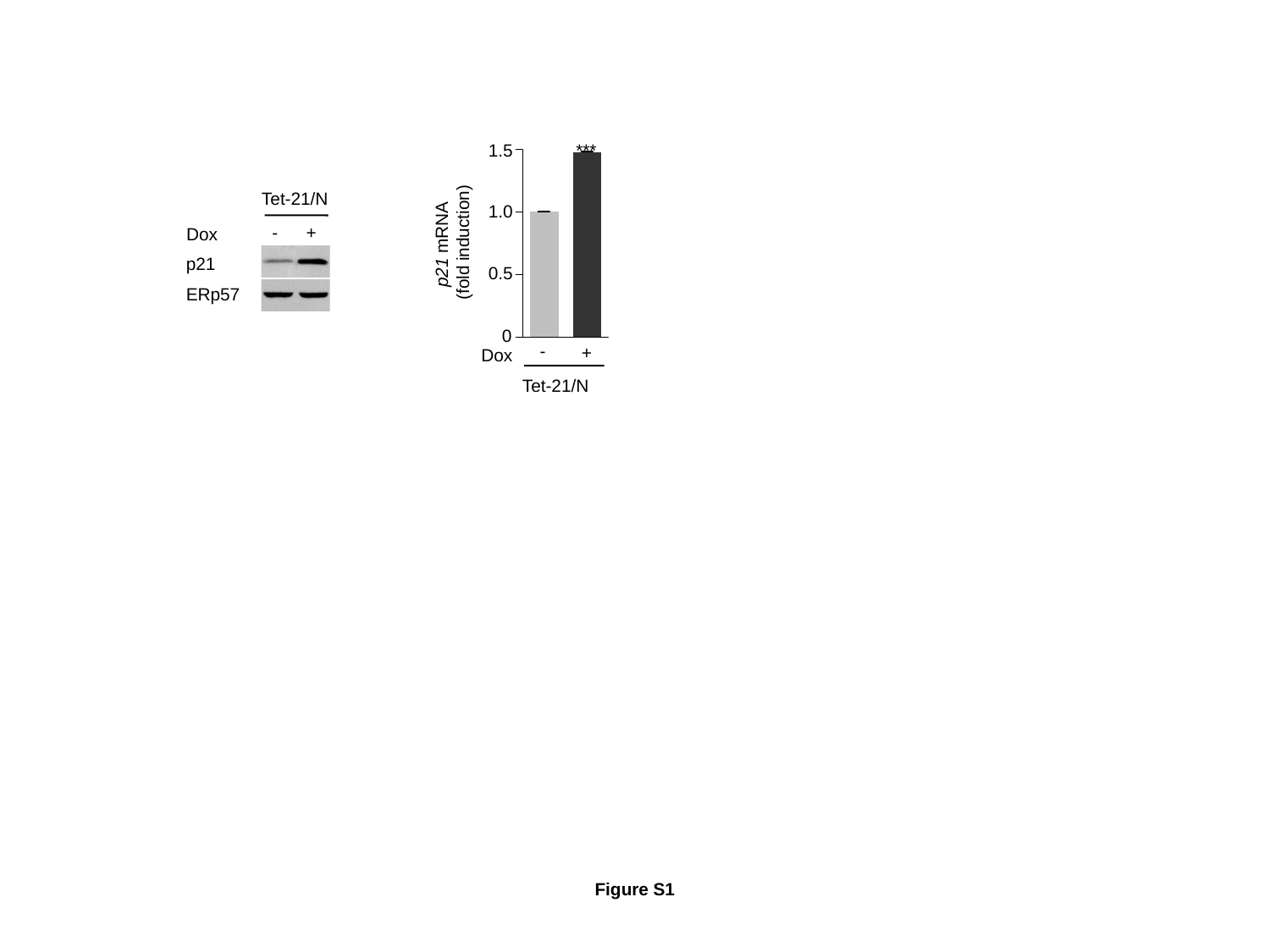

1.5
***
Tet-21/N
-
+
Dox
p21
ERp57
1.0
p21 mRNA
(fold induction)
0.5
0
-
+
Dox
Tet-21/N
Figure S1

Supplement: Figure S1 — p21 is upmodulated in Tet-21/N cells upon treatment with doxycyclin Western blot (left panel) and qRT-PCR (right panel) of p21 expression in Tet-21/N untreated or treated with doxycyclin for 96 h. A representative Western blot is shown. Mean±s.d. (n = 3). *** P<0.001. (0.23 MB PPT) [file pone.0002236.s001.ppt]
